# Autophagy induction by *Mycobacterium indicus pranii* promotes *Mycobacterium tuberculosis* clearance from RAW 264.7 macrophages

**DOI:** 10.1371/journal.pone.0189606

**Published:** 2017-12-13

**Authors:** Bindu Singh, Mohd Saqib, Ananya Gupta, Pawan Kumar, Sangeeta Bhaskar

**Affiliations:** 1 Product Development Cell, National Institute of Immunology, New Delhi, Delhi, India; 2 Special Centre for Molecular Medicine, Jawaharlal Nehru University, New Delhi, India; Indian Institute of Technology Delhi, INDIA

## Abstract

*Mycobacterium indicus pranii* (MIP) is a potent vaccine candidate against tuberculosis (TB) as it has demonstrated significant protection in animal models of tuberculosis as well as in clinical trials. Higher protective efficacy of MIP against TB as compared to BCG provoked the efforts to gain insight into the molecular mechanisms underlying MIP mediated protection against *Mycobacterium tuberculosis* (M.tb). Autophagy, initially described as a cell survival mechanism during starvation, also plays a key role in host resistance to M.tb. Virulent mycobacteria like M.tb, suppresses host autophagy response to increase its survival in macrophages. Since mycobacterial species have been shown to vary widely in their autophagy-inducing properties, in the present study, we examined the autophagy inducing efficacy of MIP and its role in MIP-mediated protection against M.tb. MIP was found to be potent inducer of autophagy in macrophages. Induced autophagy was responsible for reversal of the phagosome maturation block and phagolysosome fusion inhibition in M.tb infected macrophages, which ultimately lead to significantly enhanced clearance of M.tb from the macrophages. This is an important study which further delineated the underlying mechanisms for significant immunotherapeutic activity observed in TB patients / animal models of tuberculosis, given MIP therapy along with chemotherapy.

## Introduction

Tuberculosis (TB), a chronic infectious disease, remains one of the world’s deadliest communicable diseases (WHO report 2016). *Mycobacterium tuberculosis* (M.tb), the causative organism of TB, is a highly successful pathogen as it has evolved number of survival strategies to evade the host immune mechanisms [1]. Blocking of phagosome maturation and phago-lysosome fusion, interference with antigen presentation, resistance to reactive oxygen and nitrogen intermediates [2,3], alteration of host cell apoptotic pathways [4] and inhibition of autophagy in host cells [5] are some of the strategies which enhance M.tb survival inside the macrophages.

Autophagy, a lysosomal degradation pathway which contributes to maintenance of intracellular homeostasis, has been shown to be an integral part of both adaptive and innate immunity [6]. It was initially known as stress response involved in cell survival during nutrient starvation condition and for its role in maintaining intracellular homeostasis, by eliminating surplus or damaged organelles and degradation of superfluous, misfolded and damaged proteins [7]. Subsequent studies showed the innate defence role of autophagy against invading pathogens including M.tb [8–10]. One of the important survival strategies of M.tb is to evade acid hydrolases by inhibiting phagosome-lysosome fusion [11,12]. Autophagy induction by physiological, pharmacological or immunological means was found to reduce intracellular M.tb survival by targeting it for lysosomal degradation [13]. The importance of autophagy genes in restricting intracellular growth of M.tb was confirmed by genome-wide small interfering RNA (siRNA) screenings in which 74 target genes were knocked down using target-specific siRNAs and their effect on M.tb survival was tested. Out of the 74 genes tested, 44 were found to be responsible for autophagy mediated M.tb clearance [14]. Studies in autophagy-deficient mice demonstrated that autophagy confers protection against active tuberculosis by reducing bacterial burden and inflammation [5,15].

*Mycobacterium indicus pranii* (MIP) is a progenitor of *M*. *avium* complex, sharing cross-reactive antigens with M.tb and *M*. *leprae*. Studies in mouse and guinea pig models of TB have shown that prophylactic or therapeutic administration of MIP leads to significant reduction in bacillary burden. MIP treated animals were found to exhibit significantly reduced TB pathology and enhanced survival [16–18]. Recently concluded multicentric clinical trial in category-II TB patients further confirmed its immunotherapeutic role in difficult to treat patients having advanced disease [19].

It has been shown previously that different mycobacterial species vary widely in their autophagy inducing potential [20]. Although MIP has shown potent anti-TB efficacy [16–18], and has been uniquely placed between slow- and fast-growing mycobacteria[21]; autophagy-inducing potential of MIP and its role in MIP-mediated protection against TB remained unexplored. The present study provides evidence that MIP is a potent inducer of autophagy in macrophages. Induced autophagy is responsible for the reversal of phagosome maturation block and phagolysosome fusion inhibition in M.tb infected macrophages, which ultimately leads to significantly enhanced clearance of M.tb from the macrophages. This study enhances our understanding of the mechanism behind MIP mediated protection in M.tb infection.

## Materials and methods

### Bacterial culture

MIP and M.tb-H37Rv were cultured in Middlebrook 7H9 broth (0.05% Tween-80, 0.1% Glycerol, supplemented with 10% Albumin Dextrose Catalase). GFP-expressing M.tb was prepared by electroporating H37Rv with pSC301 plasmid (AddGene, #31851). Transformed bacilli were selected and maintained using 7H9 broth supplemented with Hygromycin (100 μg/ml).

### Macrophage culture and infection

RAW 264.7 cells were obtained from ATCC and cultured in RPMI-1640 medium supplemented with 10% foetal bovine serum and 1 X Penicillin-Streptomycin solution. Prior to the day of experiment, cells were scraped, counted and seeded at a density of 5 x 10^5^, 1 x10^6^ and 2 x 10^6^ cells per well in 24-, 12- and 6-well plates respectively and left overnight at 37°C in CO_2_ incubator for optimal adherence. MIP culture was grown till the OD_600_ reaches 0.6–0.8 and harvested by centrifuging at a speed of 1000 g for 10 min followed by washing with Phosphate buffered saline (PBS). Single cell suspension of MIP / M.tb was prepared and OD_600_ was determined. Adhered macrophages were infected with mycobacteria (MOI of 1:10) and active infection was allowed to proceed for 4 hrs, and after that the cells were rinsed with PBS. Macrophages were harvested at the indicated time points and lysed on ice using M-2 lysis buffer (1M Tris pH 7.4; 5M NaCl; Glycerol; 10% Triton-X-100; 0.5M EDTA; 0.5M EGTA) containing 1 X PIC (Protease Inhibitor Cocktail). Lysates were then analysed for LC3 (Microtubule-associated proteins 1A / 1B light chain 3B) expression level by Western blot. For M.tb survival studies, GFP expressing M.tb (with Hygromycin as selection antibiotic) was used to infect the macrophages.

### Preparation of RAW-GFP-LC3 cells

RAW 264.7 cells were transfected with pEGFP-LC3 (AddGene, #21073) using Lipofectamine-2000 (Thermo-Fischer Scientific) as per manufacturer’s instructions. The stably transfected cells were selected using G418 antibiotic (1 mg/ml) for 15 days. Expression of GFP-LC3 was checked using flow-cytometry. When GFP-LC3 is recruited on autophagosomes, they appear as punctate structures. Briefly, GFP-LC3 expressing RAW 264.7 macrophages were seeded on sterile coverslips and allowed to adhere before being infected with MIP / M.tb and treated with Rapamycin / Bafilomycin for 4, 8, 12, 16 and 24 hrs. Cells were fixed and visualised for puncta formation by confocal microscopy.

### Western blotting

The protein content of the cellular lysates was determined using Bicinchoninic Acid (BCA) method. 20–40 μg of the protein samples were loaded on SDS-PAGE gel and electrophoresed. Electrophoresed proteins were transferred on to the Polyvinylidene difluoride (PVDF) membrane using wet transfer for 2.5 hrs at 60 V. The membrane was blocked for 1 hr at RT with 5% w/v non-fat dry milk in 1 X TBST (Tris-buffered saline with 0.1% Tween-20). Subsequently, membrane was incubated overnight at 4°C with anti-LC3 primary antibody (Cat. No. #12741, obtained from Cell Signalling Technologies, dilution-1:1000) in 5% w/v Bovine Serum Albumin (BSA) in TBST. This was followed by incubation with anti-rabbit HRP antibody (Cat. No. #7074, procured from Cell Signalling Technologies, dilution-1:5000) for 1 hr at RT. The proteins on the blot were then detected using chemiluminescence method (BioRad Clarity Western ECL substrate). LC3-II expression levels (which correlates to the number of autophagosomes formed) were then quantitated using Image-J software.

### DQ-BSA degradation assay

To quantify the lysosomal activity, the self-quenched reporter substrate, Dye-quenched Bovine Serum Albumin (DQ Red BSA) was used, which upon proteolytic cleavage, produces brightly fluorescent products. This provides a measure of the overall proteolytic/ lysosomal activity within the cell. Briefly, RAW 264.7 macrophages were seeded on sterile coverslips and kept overnight for adherence. The cells were then loaded with DQ Red BSA (10 μg/ml) prepared in complete RPMI for 1 hr, followed by chasing in incomplete RPMI for 2 hrs at 37°C. The cells were then washed and infected with MIP / M.tb (MOI-1:10) or treated with Rapamycin for 4 hrs. Subsequently, cells were fixed with 4% Paraformaldehyde (PFA) for 10 min at RT and visualised by confocal microscopy or analysed by flow cytometry to quantify the DQ-BSA fluorescence.

### Analysis of phagosome maturation by immunofluorescence

Phagosome maturation in different experimental groups was analyzed by confocal microscopy. RAW 264.7 cells were seeded on sterile coverslips and kept for adherence overnight at 37°C in CO_2_ incubator. On the day of experiment, macrophages were infected with MIP at MOI of 10 and kept for 4 hrs at 37°C in CO_2_ incubator. The cultured cells were fixed in absolute methanol for 10 min at RT, followed by washing with PBS thrice. The cells were then permeabilized with 0.1% Triton-X-100 in PBS for 10 min at RT. Blocking was done for 2 hrs at RT with buffer containing 3% BSA and 0.1% Triton-X-100 in PBS. Cells were washed 3 times with PBS. This was followed by incubation with Rab5 (Cat. No. #3547) / Rab7 (Cat. No. #9367) primary antibodies (obtained from Cell Signalling Technologies, dilution-1:100) in blocking buffer at 4°C for overnight. Cells were again washed and Alexa fluor 488 secondary antibody (Cat. No. #A-21206, Life technologies; dilution-1:1000) in blocking buffer was then added and incubated for 45 to 60 min at RT. After washing 3 times with PBS, DAPI (4′,6-Diamidine-2′-phenylindole dihydrochloride) staining was done (dilution-1:500) for 5 min. Washing was done once with PBS for 5 min. The coverslips were then dried and mounted on the slides with anti-fade mounting media and observed by Zeiss LSM 510 Meta Confocal Microscope at 63 X magnification.

### Assessment of M.tb phagosome-lysosome fusion

RAW macrophages, plated on sterile coverslips were infected with GFP expressing M.tb (MOI-1:10) for 4 hrs. After establishment of the M.tb infection, extracellular bacteria were washed out and the macrophages were co-infected with MIP for 4 hrs. The cultured cells were then fixed in 4% PFA and permeabilized with 0.1% Triton-X-100 in PBS for 10 min at RT. Subsequently, the cells were stained with LysoTracker Red (100 nM) for 30 min at 37°C and washed with PBS to remove excess dye. DAPI staining was done and co-localization of GFP expressing M.tb with LysoTracker Red was observed by confocal microscope. Fifty fields of each group were examined and total number of green spots (GFP expressing M.tb located outside the lysosomes) and yellow spots (formed by co-localisation of GFP expressing M.tb with LysoTracker Red giving a yellow fluorescence) were counted.

### Evaluation of M.tb survival

RAW 264.7 cells were infected with GFP expressing M.tb H37Rv for 4 hrs at MOI 1:10. The extracellular M.tb was washed out after the establishment of infection and the cells were co-infected with MIP. After 4 hrs, the cells were again washed with PBS and lysed for 15 min with 0.1% Triton-X-100 in PBS. 100 μl lysate from each group was plated in triplicates on 7H11-agar plates containing Hygromycin as the selection antibiotic for GFP expressing M.tb. Colonies of M.tb were counted after 24–28 days of growth at 37°C and percentage survival of M.tb in different experimental groups was analysed.

### Autophagy abrogation by RNA interference technique

RAW 264.7 macrophages were harvested and seeded at a density of 0.5 x 10^6^ per well in 6-well plate and kept for 24 hrs at 37°C in CO_2_ incubator for optimal adherence. Prior to transfection, 50 ng of LC3 SiRNA was diluted in 500 μl of Opti-MEM and added to RNAiMax transfection reagent. The contents were mixed well and incubated for 5 min at RT. The SiRNA-RNAiMax mixture was then added drop-wise to the wells. Cells were incubated in CO_2_ incubator for 6 hrs after which, the transfection mixture was replaced with fresh complete media. Cells were again incubated for 24 hrs and then used for experiments. Given below is the sequence of SiRNA used in this study:

LC3 –GCGAGUUGGUCAAGAUCAUdTdT

### Statistical analysis

The difference in the variables was analyzed by one-way analysis of variance (One-way ANOVA). P-value <0.05 was considered to be statistically significant.

## Results

### MIP is a potent autophagy inducer in macrophages

Since different mycobacterial species vary in their autophagy inducing potential, first we examined the efficacy of MIP for induction of autophagy in RAW 264.7 macrophage cell line. Cellular lysates prepared from MIP- infected RAW macrophages were probed for LC3-I / II expression by western blotting. The LC3 protein, a specific constituent of the autophagosomal membrane, is the only highly specific marker of the autophagosome presently [22]. It is known to exist in two forms: LC3-I (the 18 kDa cytosolic form) and LC3-II (formed upon conjugation of LC3-I to PE has molecular weight of 16 kDa). LC3-II is recruited to the inner as well as outer autophagosomal membranes [23]. Quantification of LC3-II correlates with the extent of autophagy induced.

**Fig 1:**
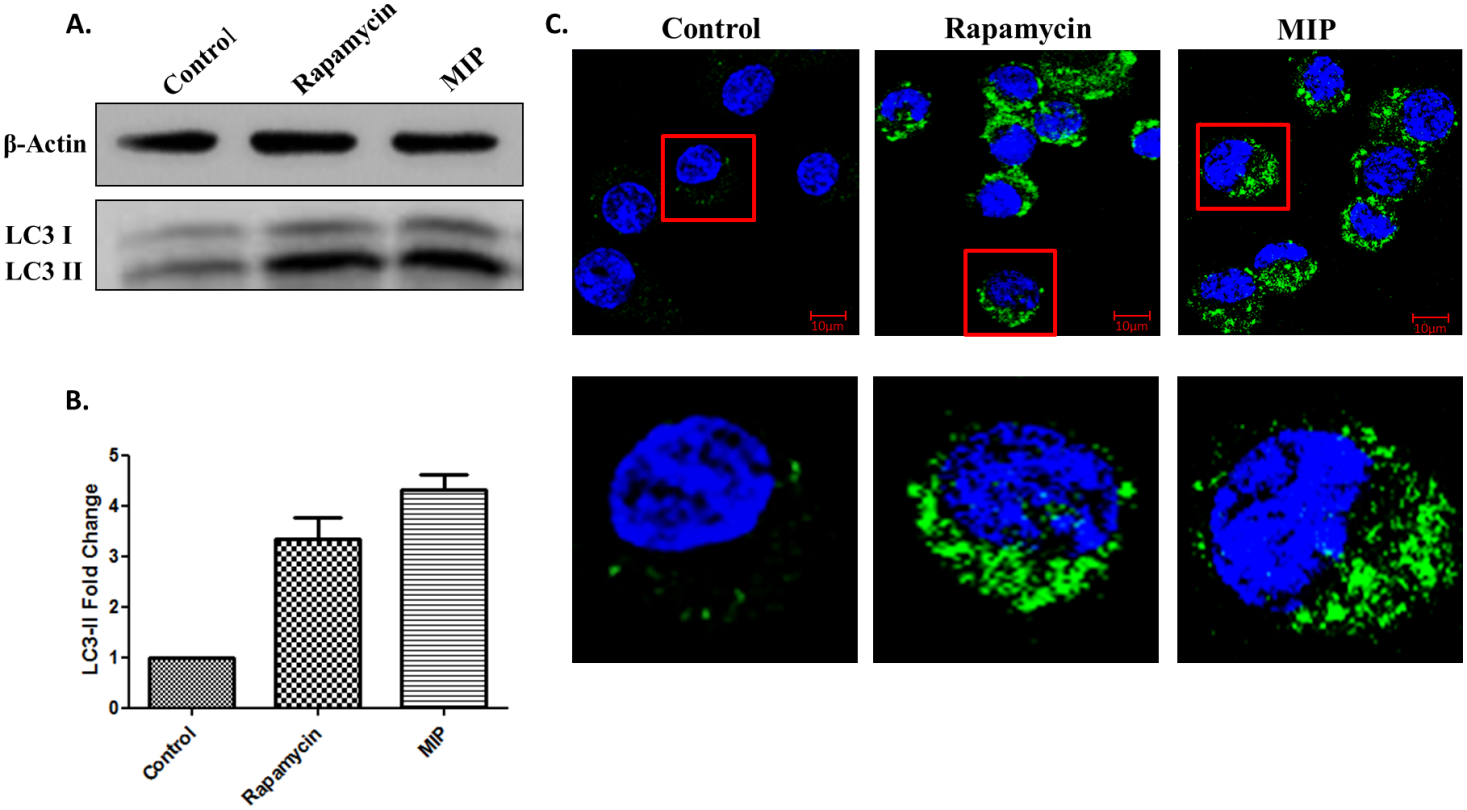
MIP is a potent inducer of autophagy. RAW 264.7 macrophages were infected with MIP (MOI-1:10) for 4 hrs, whole cell lysates were prepared and western blot was performed. **(A)** Shown is the representative blot depicting the level of lipidated LC3-II in Control, Rapamycin treated and MIP infected macrophages. **(B)** Shown is the mean fold change ± range in LC3-II level in Rapamycin treated and MIP infected cells as compared to uninfected control. **(C)** RAW cells expressing GFP-LC3 were infected overnight (MOI-1:10) with MIP. Images were taken at 63 X magnification. Shown are the merged images of GFP-LC3 (green) and DAPI (blue). ***: P<0.0001.

It was observed that MIP-infected macrophages expressed 4.3 ± 0.3 fold higher levels of LC3-II compared with uninfected cells (Fig 1A and 1B). Rapamycin was used as a positive control. During autophagy, LC3-I is recruited to the inner membrane of autophagosomes and is converted to LC3-II. Higher level of LC3-II in MIP- infected RAW 264.7 cells suggested autophagy induction in them.

To confirm the ability of MIP to induce autophagy, infected macrophages were examined for puncta formation by confocal microscopy. GFP-LC3 expressing cells were used for this assay. Large and numerous puncta were seen in MIP-infected cells. We observed markedly enhanced puncta formation in MIP-infected macrophages, compared with uninfected cells (Fig 1C). Rapamycin, used as positive control, also induced puncta formation in macrophages.

### Total autophagic flux is maintained in MIP infected macrophages

**Fig 2:**
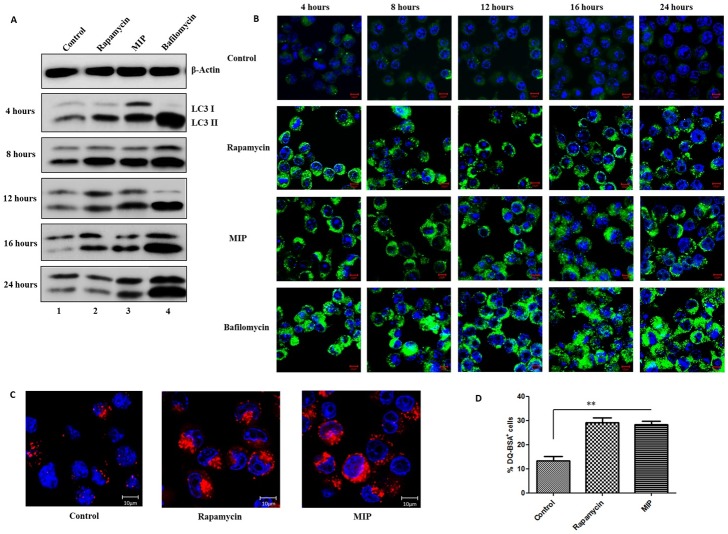
Total autophagic flux is maintained in MIP infected macrophages. RAW 264.7 macrophages were infected with MIP or treated with Rapamycin (1 μM) / Bafilomycin A1 (100 nM) and lysates were prepared at 4, 8, 12, 16 and 24 hrs and western blot was performed. Bafilomycin A1 was taken as positive control for fusion inhibition. **(A)** Western blot depicting the level of LC3-II in MIP infected and Rapamycin / Bafilomycin A1 treated cells. **(B)** RAW GFP-LC3 cells were treated with Rapamycin / Bafilomycin A1 or infected with MIP for 4, 8, 12, 16 and 24 hrs and imaging was done by confocal microscopy. Shown are the representative merged images of GFP-LC3 (green) and DAPI (blue) portraying LC3 puncta formation in various groups. **(C)** RAW 264.7 cells loaded with DQ-BSA were infected with MIP for 4 hrs and imaged at 63 X magnification. Merged images of DQ-BSA Red (red) and DAPI (blue), depicting the proteolysis of DQ-BSA are shown here. **(D)** DQ-BSA-loaded cells were infected with MIP for 4 hrs, and the percentage of DQ-BSA positive cells was determined by flow cytometry. **: P<0.001.

Under normal circumstances, autophagosomes fuse with lysosomes wherein their contents are degraded by acid hydrolases. However, certain mycobacterium species including M.tb have been shown to inhibit this step, resulting in the accumulation of autophagosomes. To examine the fate of autophagosomes in MIP- infected RAW 264.7 macrophages, these were infected with MIP for different time duration and their lysates were assessed for LC3-II. Bafilomycin A1 is a known inhibitor of autophagosome-lysosome fusion and was included as a control. Persistently higher level of LC3-II was observed in Bafilomycin A1-treated cells. Whereas, in MIP-infected cells, level of LC3-II was found to decline with time, suggesting autophagosomal fusion with lysosomes (Fig 2A). To further validate these findings, puncta formation in MIP- infected macrophages was analysed at different time points. Accumulation of autophagosomes was observed in Bafilomycin-treated cells, but not in MIP- infected cells (Fig 2B). Rapamycin was used as an experimental control.

In the last step of autophagy, contents of autophagosomes are degraded by acid hydrolases. To examine this step in MIP-infected macrophages, self-quenched reporter substrate, DQ Red BSA was used which becomes fluorescent upon proteolytic cleavage. MIP-infected, DQ BSA-loaded macrophages were examined for fluorescence signals by confocal microscopy. Higher levels of DQ Red BSA signals were noted in MIP- infected cells, compared with uninfected cells (Fig 2C). Percentage of DQ-BSA positive cells were also analysed by flow cytometry. Significantly higher percentage of DQ-BSA positive cells were observed in MIP-infected macrophages, compared with uninfected cells (Fig 2D).

**Fig 3:**
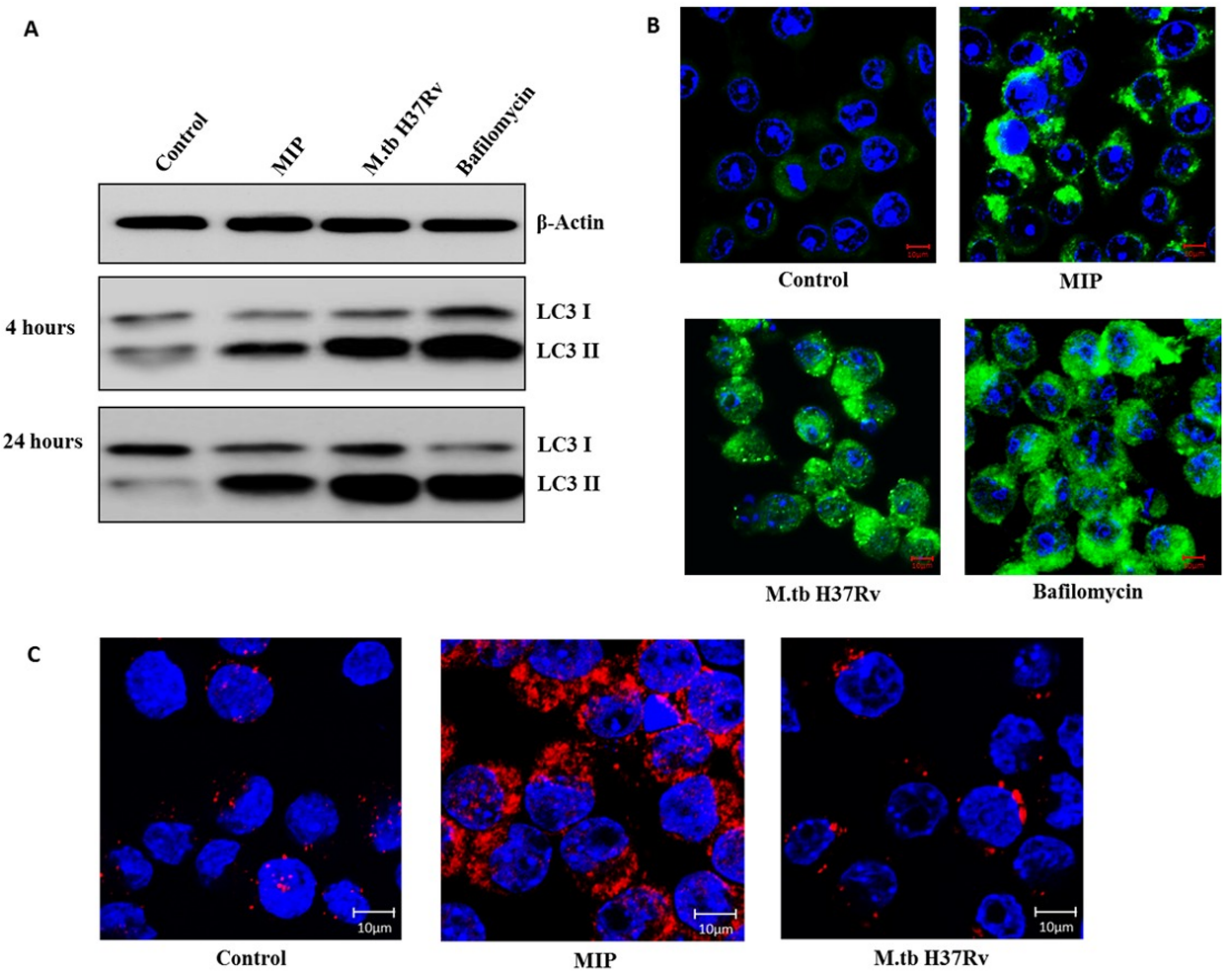
M.tb induces autophagosome formation but inhibits its fusion with lysosome and proteolysis. RAW 264.7 macrophages were infected with MIP/ M.tb H37Rv or treated with Bafilomycin A1 (100 nM) and lysates were prepared at 4 and 24 hrs followed by western blot. **(A)** Representative blot depicting LC3-II levels in MIP/ M.tb H37Rv infected and Bafilomycin A1 treated cells. **(B)** RAW GFP-LC3 cells were treated with Bafilomycin A1 or infected with MIP / M.tb H37Rv for 4 and 24 hrs and imaged by confocal microscopy. Shown are the representative merged images of GFP-LC3 (green) and DAPI (blue). **(C)** RAW 264.7 cells loaded with DQ-BSA were infected with MIP / M.tb H37Rv for 4 hrs and imaged at 63 X magnification. Shown here are the merged images of DQ-BSA Red (red) and DAPI (blue), depicting the proteolysis.

Next, we examined how MIP and M.tb differ in their autophagy-inducing properties. RAW 264.7 cells were infected with MIP and M.tb and their lysates were probed for LC3-II levels. It was observed that both MIP and M.tb led to significant expression of LC3-II in macrophages. These findings were in agreement with previous studies demonstrating the autophagosome formation in M.tb infected cells (Fig 3A). Consistent with these findings, marked puncta formation was observed in both MIP- and M.tb-infected macrophages (Fig 3B). Analysis of autophagosome-lysosome fusion using DQ Red BSA dye revealed that autophagosomes in M.tb-infected cells failed to fuse with lysosomes (Fig 3C).

### MIP-induced autophagy promotes maturation of M.tb containing phagosomes

**Fig 4:**
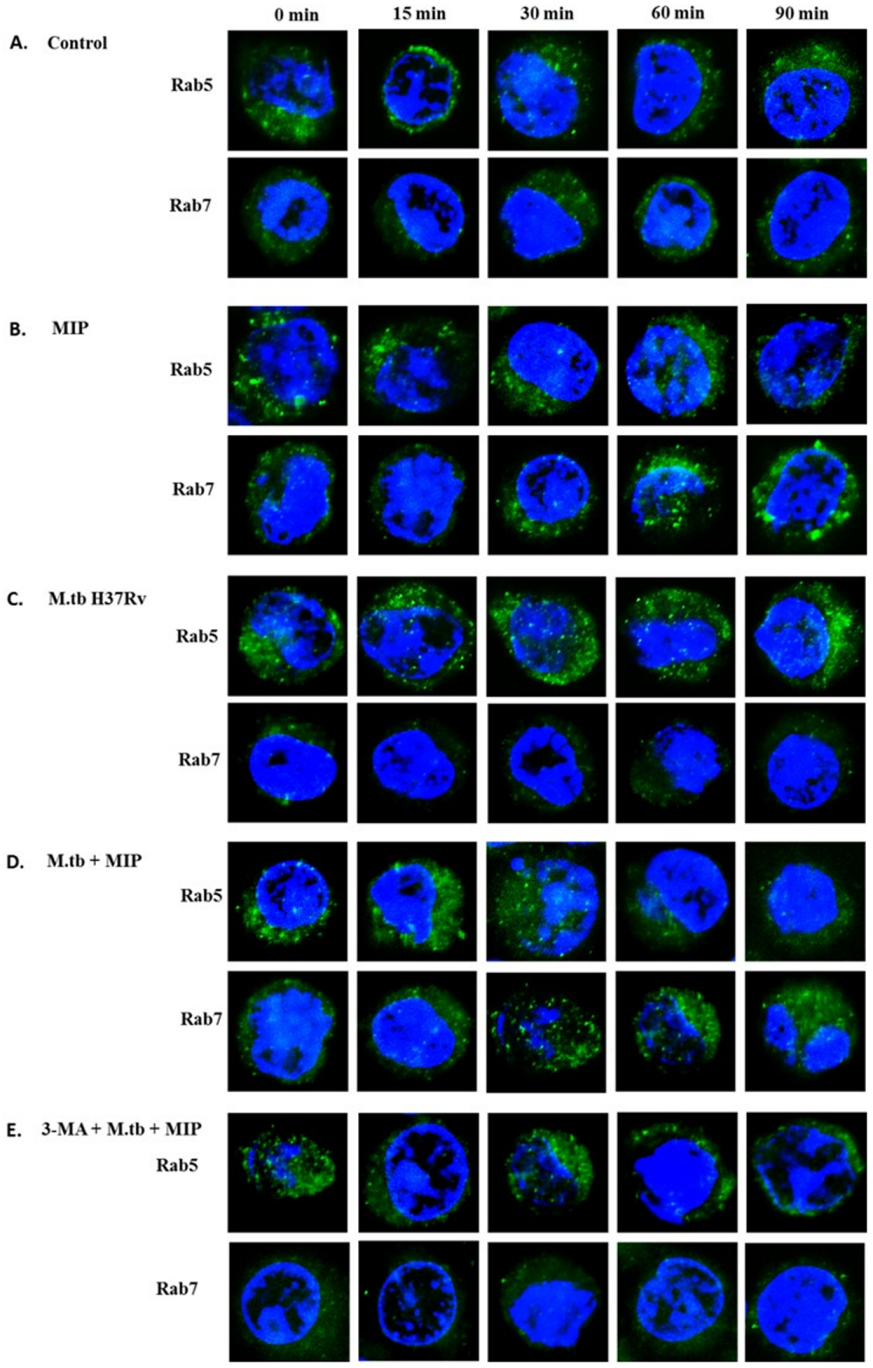
MIP promotes maturation of M.tb containing phagosomes via autophagy. Immunofluorescence images of macrophages stained for Rab5 (marker for early phagosomes) and Rab7 (marker present on late phagosomes). **(A)** Uninfected RAW 264.7 cells **(B)** RAW 264.7 macrophages infected with MIP and **(C)** with M.tb H37Rv **(D)** Co-infected with M.tb and MIP **(E)** treated with 3-MA (5 mM) for 1 hr to inhibit autophagy followed by M.tb and MIP infection. The cells were fixed and permeabilized followed by staining with Rab5 to Rab7 at different time points. Rab5 to Rab7 conversion was observed in MIP infected macrophages **(4B)** while M.tb was seen to inhibit the conversion process **(4C)**. When M.tb infected cells were re-infected with MIP, it was seen that MIP promotes maturation of M.tb containing phagosomes **(4D)**. Upon autophagy inhibition with 3-MA, maturation of M.tb containing phagosomes was inhibited even in the presence of MIP **(4E)**.

One of the important survival mechanisms of M.tb within the host is to inhibit phagosome maturation. Previous studies have shown that induction of autophagy in M.tb infected cells promotes the phagosome maturation and fusion with lysosome. Given potent autophagy inducing efficacy of MIP, we examined the effect of MIP-stimulation on phagosomal maturation. For this, MIP- infected cells were immunostained for Rab5 (a marker for early phagosomes) and Rab7 (a marker present on late phagosomes) at different time points after infection. It was observed that Rab5 to Rab7 conversion took place efficiently in MIP-infected macrophages. However, conversion of Rab5 to Rab7 was found to be inhibited in M.tb-infected cells (Fig 4B and 4C).

To investigate whether MIP co-infection in M.tb-infected cells promotes phagosome maturation, RAW 264.7 cells were first infected with M.tb H37Rv, washed with PBS to remove extracellular M.tb-H37Rv, and co-infected with MIP. Efficient conversion of Rab5 to Rab7 in these cells showed that MIP promoted the maturation of M.tb containing phagosomes (Fig 4D). To confirm the role of autophagy in MIP-induced Rab5 to Rab7 conversion in M.tb co-infected cells; macrophages were pre-treated with 3-Methyl Adenine (3-MA, an autophagy inhibitor) were infected with M.tb followed by MIP stimulation with subsequent immunostaining for Rab5 and Rab7. It was observed that pre-treatment of cells with 3-MA markedly inhibited the Rab5 conversion to Rab7 (Fig 4E). These results showed that MIP alleviates the inhibitory effect of M.tb on phagosome maturation through autophagy.

### MIP-induced autophagy promotes phagosome-lysosome fusion in M.tb-infected macrophages

**Fig 5:**
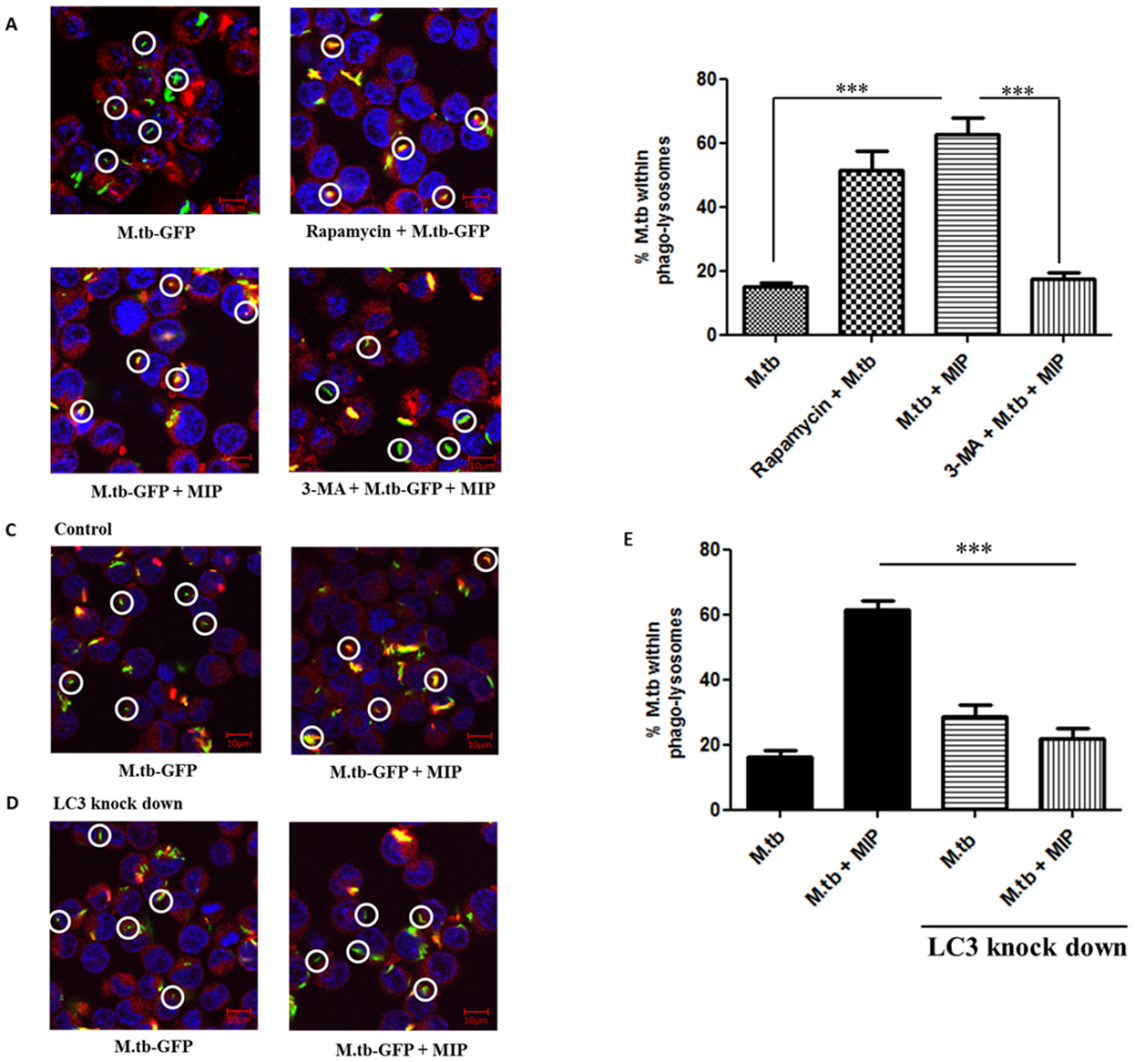
MIP induced autophagy results in enhanced phago-lysosome fusion in M.tb infected macrophages. RAW 264.7 cells were co-infected with GFP expressing M.tb and MIP followed by LysoTracker-Red staining to stain the lysosomes. Slides were prepared and visualised by confocal microscopy to examine the co-localisation of GFP expressing M.tb within the lysosomes. 50 fields of each group were examined and green spots (M.tb located outside the lysosomes) and yellow spots (formed by co-localisation of GFP expressing M.tb with LysoTracker-Red giving a yellow fluorescence) were counted. **(A)** Confocal images showing co-localisation of GFP expressing M.tb within the lysosomes in different groups. **(B)** Graphical representation of the percentage of GFP expressing M.tb present within the lysosomes. **(C)** Representative images depicting the co-localisation of GFP expressing M.tb within the lysosomes of RAW 264.7 cells with basal level of autophagy. **(D)** Representative images showing the co-localisation of GFP expressing M.tb within the lysosomes of RAW 264.7 cells in which LC3 was knocked down using RNA interference technique. **(E)** Graphical representation of the percentage of GFP expressing M.tb present within the lysosomes after autophagy abrogation. ***: P<0.0001.

Effect of MIP on M.tb-mediated inhibition of phagosome-lysosome was examined. For this, RAW 264.7 cells were infected with GFP-expressing M.tb. After treatement with MIP, these cells were stained with LysoTracker Red and examined for localisation of bacilli within the lysosomes (co-localised GFP-expressing M.tb and LysoTracker Red would result in yellow fluorescence signal). In ‘only M.tb’ infected cells, most of the signals observed were green indicating the localization of M.tb within the phagosomes, which did not fuse with lysosomes (Fig 5A). However, marked yellow signals were observed in the ‘M.tb + MIP’ group which suggested fusion of M.tb containing phagosomes with lysosomes in these cells (Fig 5B). Localisation of GFP-expressing M.tb within lysosomes was quantified and it was found to be 62.8 ± 5% in ‘M.tb + MIP’ group and 15.1 ± 1.2% in ‘only M.tb’ group (**Fig B and C in** S1 File).

To confirm the role of autophagy in MIP-mediated phagosome-lysosome fusion in M.tb-infected RAW 264.7 cells, the above experiments were repeated in the presence of 3-MA. Treatment of cells with 3-MA resulted in comparable yellow fluorescent signals in ‘only M.tb’ and ‘M.tb + MIP’ group **(**Fig 5B; **Fig B and C in** S1 File). These results were further confirmed by knocking down LC3 by RNA interference (**Fig A in** S1 File). A significant reduction in yellow fluorescent signal was observed in ‘M.tb + MIP’ co-infected macrophages with abrogated LC3 (% co-localisation dropped to 21.8 ± 3.4% from 61.2 ± 2.8%) (Fig 5C–5E; **Fig A and B in** S2 File). These results confirmed that MIP promotes phagosome-lysosome fusion in M.tb-infected macrophages by inducing autophagy in them.

### MIP-induced autophagy enhances M.tb clearance from infected macrophages

Above findings provide evidence that MIP induced autophagy overcomes the mycobacterial block of phagosome maturation and targets the M.tb containing phagosomes to the lysosomes. Final concluding step was to investigate if MIP induced autophagy can affect M.tb survival in infected macrophages. The effect of autophagy induction by MIP on M.tb viability was analysed by direct scoring of colony-forming units of M.tb.

**Fig 6:**
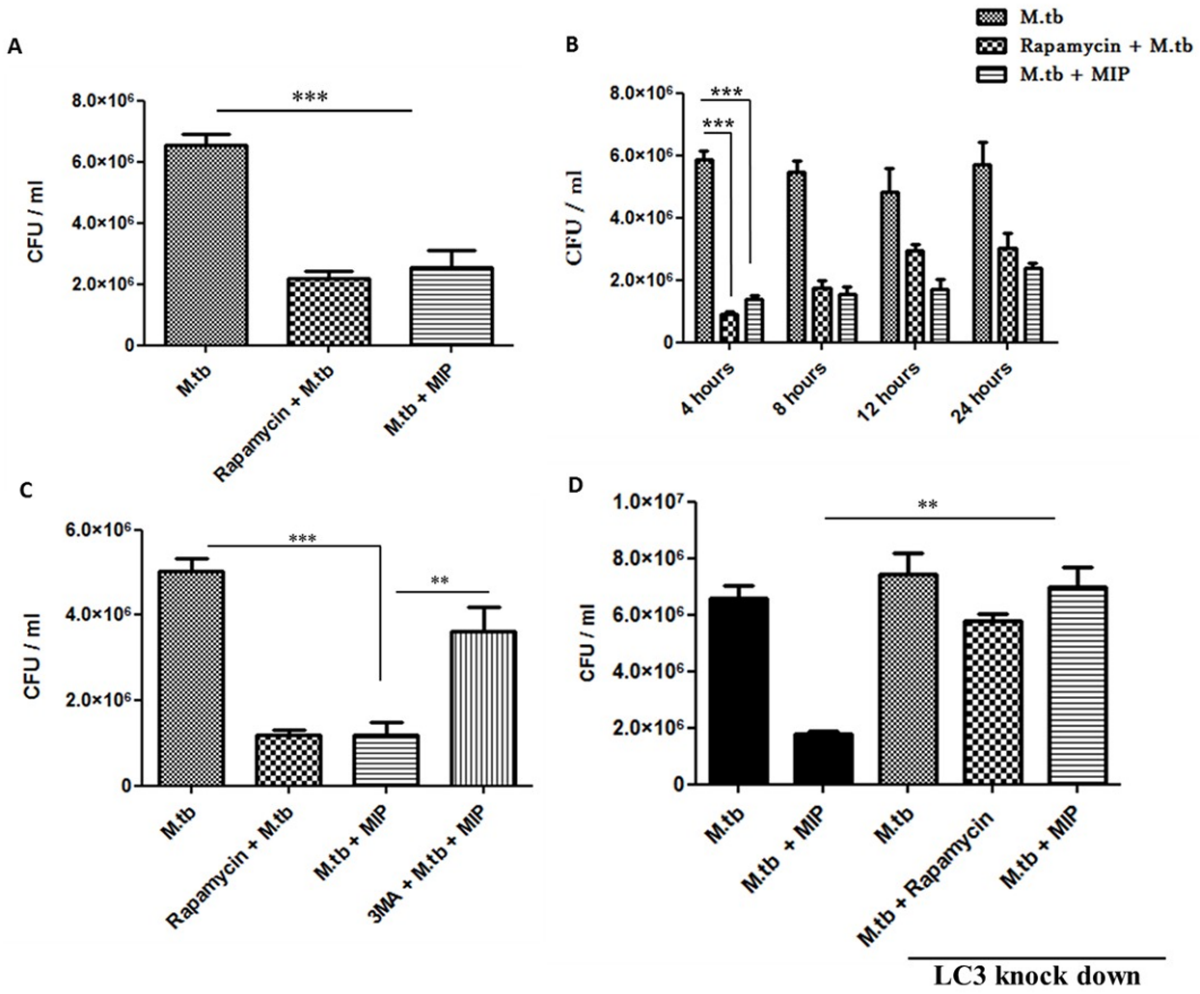
Autophagy induction by MIP enhances clearance of M.tb from infected macrophages. **(A)** RAW 264.7 macrophages were infected with either GFP expressing M.tb or co-infected with GFP expressing M.tb and MIP for 4 hrs followed by lysis. 100 μl of lysate from each group was plated in triplicates on 7H11-agar plates containing Hygromycin. Shown is the mean M.tb CFU count of 4 independent experiments. **(B)** Macrophages were infected with either M.tb or co-infected M.tb and MIP for 4, 8, 12, 24 hrs and lysed followed by plating in triplicates on 7H11-agar plates containing Hygromycin. Shown is the mean M.tb CFU count of 3 independent experiments. **(C)** RAW macrophages were treated with 3-MA for 1 hr to inhibit autophagy followed by M.tb and MIP infection. Graph showing CFU count of M.tb upon autophagy inhibition. **(D)** Autophagy was abrogated using SiRNA against LC3 and the same assay was performed. It was observed that when autophagy was abrogated by loss of LC3 protein, survival of M.tb was increased. This concludes that MIP induced autophagy plays an important role in M.tb clearance. ***: P<0.0001, **: P<0.001.

Macrophages were infected with GFP expressing M.tb followed by MIP infection. The cells were lysed and plated on 7H11-agar plates containing Hygromycin as selection antibiotic for GFP-expressing M.tb. The number of viable M.tb in the group which was co-infected with MIP was significantly less as compared to control group (only M.tb infected macrophages). The percentage of viable M.tb in MIP co-infected macrophages reduced to 31 ± 5.4% from 100% in ‘alone M.tb’ infected group. The reduction in the number of viable M.tb in MIP co-infected group was comparable to that of Rapamycin treated group (33 ± 2.3%) (Fig 6A).

Next, to confirm the role of MIP induced autophagy in clearance of M.tb, autophagy inhibitor (3-MA) was used along with MIP in M.tb infected macrophages. We found that, inhibition of autophagy by the pharmacological inhibitor, prevented clearance of M.tb. In this group, the percentage of viable M.tb increased to 71.6 ± 9.1 from 23.8 ± 6.3 (M.tb + MIP co-infected group) (Fig 6C).

To further confirm the role of MIP induced autophagy in M.tb clearance, autophagy was abrogated using SiRNA against LC3 protein. In the groups where autophagy was inhibited by loss of LC3 protein, M.tb clearance from the macrophages was significantly reduced even in the presence of MIP (Fig 6D). In conclusion, these results delineate the underlying mechanism of MIP immunotherapy in M.tb infected host. MIP induced autophagy could be playing major role besides other mechanisms (viz. induction of Th1 and Th17 immune response) in reduction of M.tb load when MIP immunotherapy is given by aerosol route in infected lungs.

## Discussion

The host-mycobacterial interaction is a complex phenomenon, which lead to different outcomes ranging from clearance of bacteria from the host cells to cause latent infection. Although a multitude of factors are involved, the process of autophagy is widely recognized in determining the course of infection. Autophagy plays an integral role in both innate and adaptive immunity to various pathogens [24]. It is well established that in eukaryotic cells, autophagy is one of the most powerful and efficient ways of elimination of intracellular pathogens, including M.tb [8,25]. In order to prevent their clearance from the host cells and to establish persistent infection, these microbes have evolved various strategies to evade the autophagic machinery of these cells [25–27]. In this study, behaviour of MIP in the context of autophagy was investigated, which is placed in between the saprophytic and pathogenic mycobacterial species. MIP possesses a unique immunological significance. Owing to its established immunomodulatory potential in human diseases including TB and leprosy, MIP is treated as the potential vaccine candidate for both of these diseases. Previously, our group has shown immunotherapeutic efficacy of MIP given by aerosol route in M.tb infected animals [16,17]. The present study was done to gain insight into the mechanisms by which MIP decreases M.tb load in the infected animals.

Literature suggests that autophagy induction varies with mycobacterial species. Species including *M*. *smegmatis* and *M*. *fortuitum* are known to induce high autophagy responses whereas BCG, *M*. *kanasii*, and H37Ra induce low autophagic response in macrophages [20]. M.tb induces significantly high autophagosome formation but inhibits autophagic pathway at the stage of acidification of autophagosomes [11] and their fusion with lysosomes [28,29]. In this study, we found that MIP, a mycobacterial species which has been uniquely placed in between slow and fast growers [21], is a potent inducer of autophagy in macrophages. It was able to induce significantly high amount of autophagy as well as maintained the autophagic flux. The autophagy induced was significantly higher as compared to control and comparable to that induced by Rapamycin (Fig 1A and 1B). Interestingly, it was noticed that M.tb H37Rv was able to induce autophagy to the same/ greater extent as MIP, but, the fusion of autophagosomes with the lysosomes was inhibited in M.tb infected macrophages while MIP allowed the fusion process. This was depicted by the consistent LC3-II level observed at different time points in MIP infected macrophages while there was accumulation of LC3-II in M.tb infected macrophages with increasing time (Fig 3A). This observation was consistent with published reports which suggest that virulent strains of M.tb impair autophagy at the level of autophagosome-lysosome fusion [30]. Host cells possess degradation mechanisms to antagonize invading pathogens whereas, virulent bacteria like M.tb modulates these systems to escape degradation by host cells. In this study, it was observed by DQ-BSA degradation assay that lysosomal proteolysis in MIP infected macrophages was significantly higher than M.tb infected cells (Fig 3C).

M.tb promotes its survival in infected host cells by blocking the maturation of the phagosomes in which it resides; which in turn results in the failure of bacilli to reach lysosomes [12,31–33]. Reports have suggested that autophagy acts as an immune effector mechanism, resulting in phagosomal maturation that mediates mycobacteria clearance [13]. Our study revealed that MIP induced autophagy overcomes the phagosome maturation block imposed by M.tb (Fig 4). Another crucial observation was that while M.tb failed to reach the lysosomes in ‘only Mtb’ infected macrophages; whereas, MIP co-infection resulted in significantly increased fusion of M.tb containing phagosomes with lysosomes. This was illustrated by increased co-localisation of GFP-expressing M.tb with the lysosomes and this enhanced co-localisation was attributed to the autophagy induced by MIP in M.tb infected macrophages (Fig 5). Autophagy is known to activate the host immune effector response against M.tb infection. M.tb is a successful intracellular pathogen that has evolved successful strategies for evading the host defenses by arresting phagosome maturation and inhibiting later stages of autophagic pathway in infected host cells. In this study, we have observed that MIP overcomes the autophagic pathway inhibition. One of the possible reason by which MIP overcomes this block might be due to generation of strong Th1 type of response along with higher expression of IFN-γ, TNF-α in the lungs of M.tb infected animals [17]. Recent studies have provided insight into the role of these cytokines in autophagy activation, thereby promoting antimycobacterial immune defenses. IFN-γ has been reported to be involved in the autophagic control of M.tb [34]. Apart from this, Mannose-lipoarabinomannan (LAM) of M.tb is known to cause phagosome maturation arrest [35]. Parallel studies done in our lab have shown that LAM isolated from MIP possesses immuno-stimulatory properties (data not shown). Therefore, possibly MIP cell wall components including LAM might be contributing towards overcoming the phagolysosome fusion block in M.tb and MIP co-infected macrophages. Furthermore, as MIP does not inhibit phagolysosome fusion; this is an important factor in maintaining the total autophagic flux in M.tb and MIP co-infected macrophages.

The activation of autophagy pathway, induced by physiological, pharmacological or immunological means, results in the elimination of intracellular M.tb by targeting it to the lysosomes [13,36]. Our results provide evidence that MIP could efficiently reduce the survival of M.tb inside the macrophages by inducing autophagy. This was confirmed by using pharmacological inhibitors of autophagy as well as by LC3 knock down. In autophagy deficient macrophages, even in the presence of MIP, the survival of M.tb was similar to control group i.e. ‘only M.tb infected macrophages’ (Fig 6).

This study enhances our understanding of the mechanism behind MIP mediated protection in M.tb infection. To the best of our knowledge, our work is the first to examine the impact of MIP infection on autophagic process in macrophages and its role in M.tb clearance. In conclusion, this study provides evidence that MIP induces significant amount of autophagy without any added influence of inducing agents. The autophagy induced by MIP in macrophages resulted in clearance of M.tb from the infected cells.

Additional studies to dissect how MIP interacts with the host autophagy machinery, are needed to be done. Understanding the interactions of MIP within the host cells will allow us to delineate the molecular pathways involved in MIP induced autophagy.

## Supporting information

S1 File **Fig A: Confirmation of LC3 knock down upon SiRNA transfection in RAW 264.7 macrophages.** Macrophages were transfected with two different concentrations of LC3 SiRNA, for 24 hrs and 48 hrs. Density values given at the bottom of the blot shows the fold change with respect to untransfected control. **Fig B: Percentage co-localisation of M.tb containing phagosomes with lysosomes was enhanced in MIP co-infected macrophages.** Shown is the data for one set of experiment. M.tb, GFP expressing *Mycobacterium tuberculosis*; MIP, *Mycobacterium indicus pranii*; 3-MA, 3-Methyl Adenine. **Fig C: Mean percentage** ± **SEM of GFP expressing M.tb present within / outside lysosomes.** (n = 3).
(TIF)

S2 File **Fig A: Percentage of M.tb co-localisation in MIP co-infected macrophages with or without autophagy abrogation.** Total number of GFP expressing M.tb, present within the lysosomes were counted and percentage was calculated. Data for one set of experiment is shown here. **Fig B: Mean percentage co-localisation** ± **SEM of GFP expressing M.tb with the lysosomes with or without autophagy abrogation.** (n = 3) ^**#**^ Control groups with basal level of autophagy, ^**$**^ Groups in which autophagy was abrogated using LC3 SiRNA.
(TIFF)

## Abbreviations

3-MA: 3-Methyl Adenine
CFU: Colony forming units
DAPI: 4′,6-Diamidine-2′-phenylindole dihydrochloride
DQ-BSA: Dye-quenched Bovine Serum Albumin
EDTA: Ethylenediaminetetraacetic acid
EGTA: ethylene glycol-bis(β-aminoethyl ether)-N,N,N’,N’-tetraacetic acid
LC3: Microtubule-associated proteins 1A/1B light chain 3B
M.tb: *Mycobacterium tuberculosis*
MIP: *Mycobacterium indicus pranii*
MOI: Multiplicity of infection
PBS: Phosphate buffered saline
PFA: Paraformaldehyde
PVDF: Polyvinylidene difluoride
RPMI: Roswell Park Memorial Institute medium
siRNA: Small interfering RNA
TB: Tuberculosis
