# Supplementary figures and images for: Autophagy induction by Mycobacterium indicus pranii promotes Mycobacterium tuberculosis clearance from RAW 264.7 macrophages
Source: PLoS One. 2017 Dec 13;12(12):e0189606. doi: 10.1371/journal.pone.0189606 (PMC5728553; doi:10.1371/journal.pone.0189606)

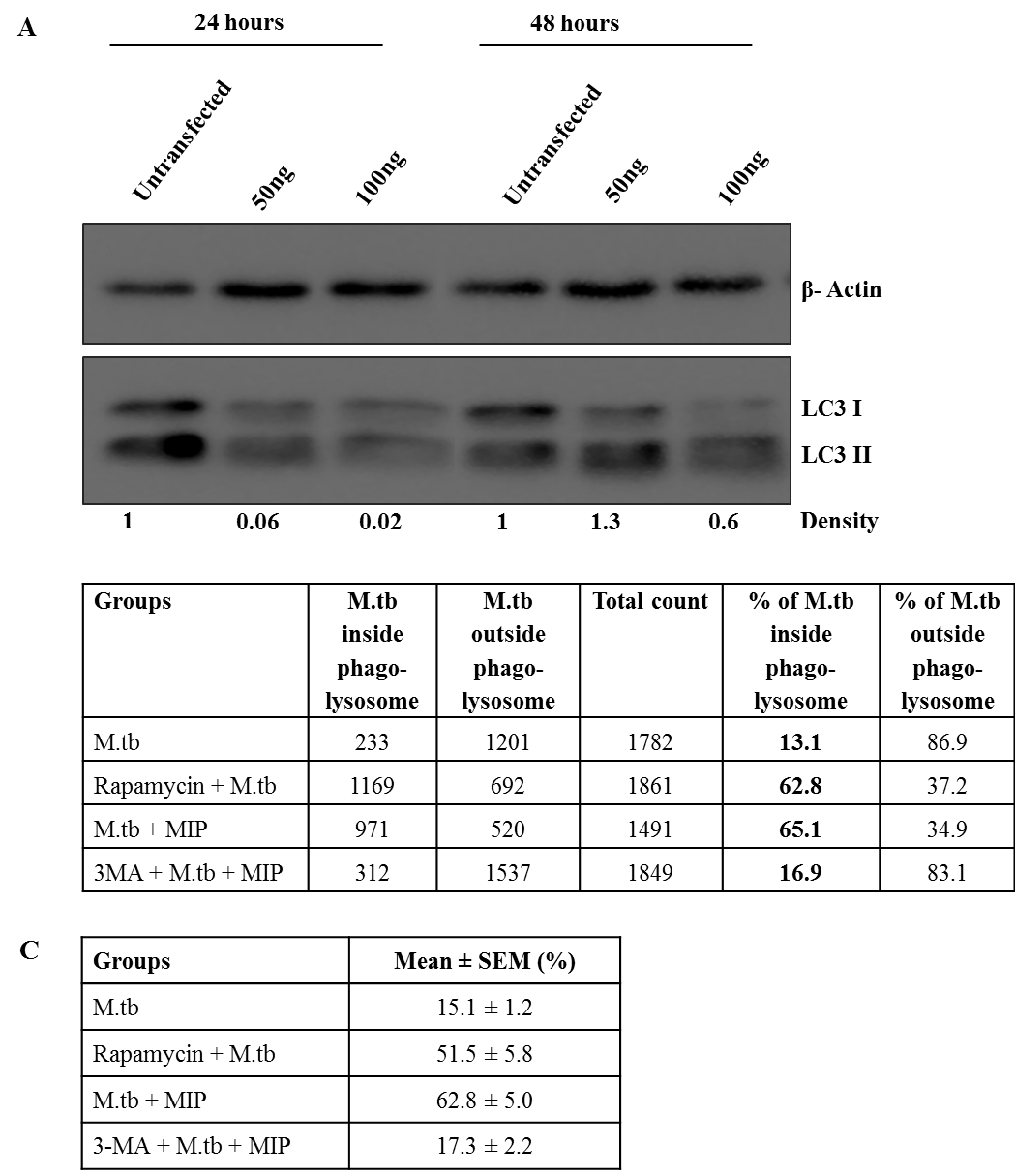

Supplement: S1 File — Fig A: Confirmation of LC3 knock down upon SiRNA transfection in RAW 264.7 macrophages. Macrophages were transfected with two different concentrations of LC3 SiRNA, for 24 hrs and 48 hrs. Density values given at the bottom of the blot shows the fold change with respect to untransfected control. Fig B: Percentage co-localisation of M.tb containing phagosomes with lysosomes was enhanced in MIP co-infected macrophages. Shown is the data for one set of experiment. M.tb, GFP expressing Mycobacterium tuberculosis; MIP, Mycobacterium indicus pranii; 3-MA, 3-Methyl Adenine. Fig C: Mean percentage ± SEM of GFP expressing M.tb present within / outside lysosomes. (n = 3). (TIF) [file pone.0189606.s001.tif]

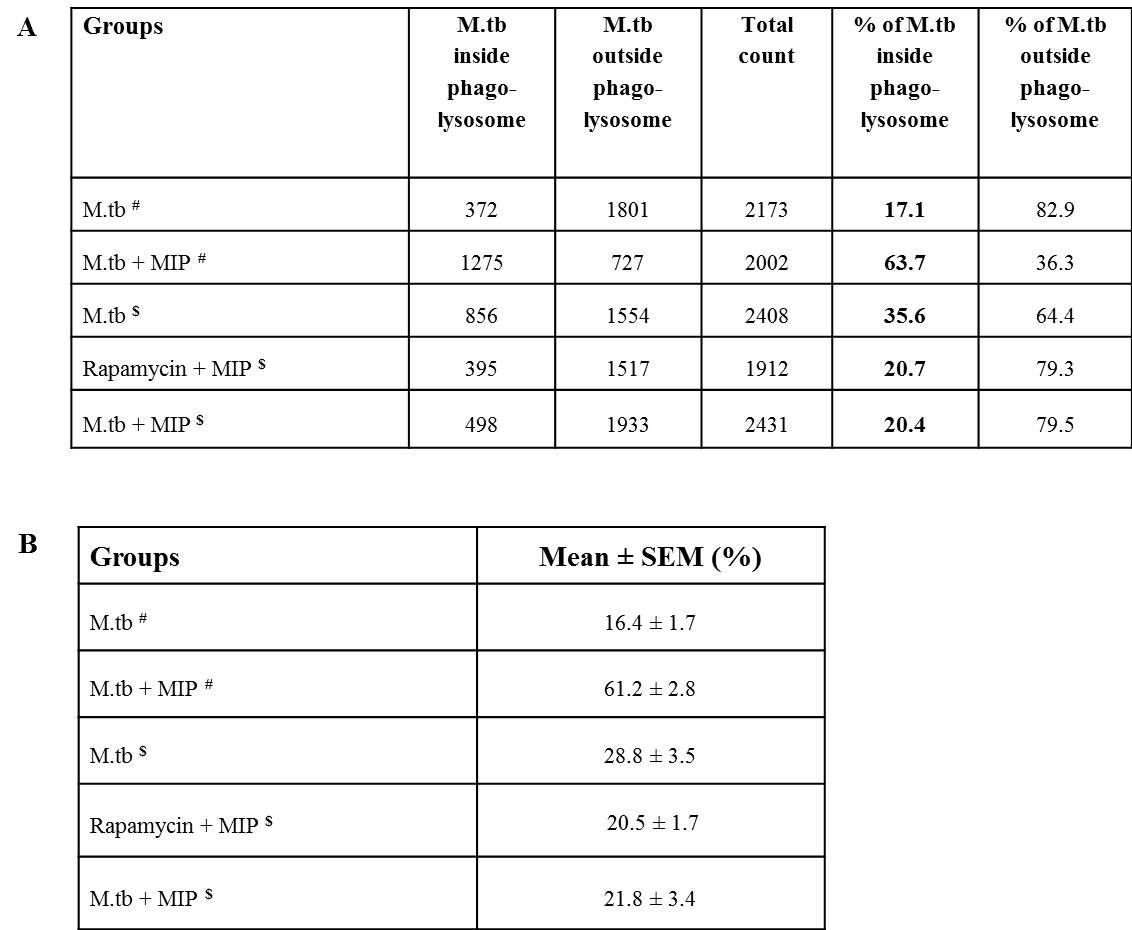

Supplement: S2 File — Fig A: Percentage of M.tb co-localisation in MIP co-infected macrophages with or without autophagy abrogation. Total number of GFP expressing M.tb, present within the lysosomes were counted and percentage was calculated. Data for one set of experiment is shown here. Fig B: Mean percentage co-localisation ± SEM of GFP expressing M.tb with the lysosomes with or without autophagy abrogation. (n = 3) # Control groups with basal level of autophagy, $ Groups in which autophagy was abrogated using LC3 SiRNA. (TIFF) [file pone.0189606.s002.tiff]
